# Report of Discussions of the Mississippi Valley Dental Association, Held March 3, 1809

**Published:** 1869-07

**Authors:** 


### Proceedings of Societies.

### REPORT OF DISCUSSIONS OF THE MISSISSIPPI VALLEY DENTAL ASSOCIATION, HELD MARCH 3, 1869.

The first subject presented for discussion, "The use of the file in operative Dentistry."

Dr. Cheesbrough: I regard the file as a very valuable instrument in operations upon the natural teeth. There are many cases in which the teeth must be separated, and the file is a valuable instrument for this. There is a very common prejudice against its use. Statistics bearing upon the effects of the use of this instrument should be kept, in order to determine the ground for such prejudice.

When the teeth are decayed I do not hesitate to file them; even when the enamel only is defective I use the file. To specify the particular conditions in which its use is indicated, would hardly be practicable now.

Dr. Jas. Taylor: I thought this question had been settled. I am aware, however, that there is still a difference of opinion, and there may be prejudice against it. For separating the teeth I advocate the free use of both the file and the wedge. We should look over the whole subject—examine it carefully. Upon some teeth we may use the file with impunity—in others it will invariably result in injury.

In some cases would file as freely as ever, in others with far more caution than twenty years ago. Front teeth slightly decayed I do not file. In lower molars would use the file freely, in some cases cutting away a large portion of the teeth. If the secretions are natural or in healthy condition, decay will not usually recur; if, however, the saliva is of an acid reaction, would use the file with far more caution. When there is defective enamel and the general recuperative power good, use the file till all the effected part is cut away and the surface smooth. I have, in some instances, in this manner cut away one-third to one-half of the crown of the tooth, with the best results. I do not believe in the practice of making contour fillings in the molars and bicuspids. I think that has given Dr. Arthur some grounds for his positions. I have lost two of my own teeth by attempts to make contour fillings. Decay often occurs on the proximal surface of the teeth, at the curvical portion. When the bicuspids, for instance, incline toward each other, I would not file; if, however, they do not so incline, I would file freely.

Dr. Morgan: I have been in the habit of using the file extensively. I use the file freely in all cases where there is disintegration of the enamel only, then polish well. I am in the habit of separating the teeth with the file. Teeth, the necks of which are in contact, I always separate with the file; in all cases separate the molars by this means. I file the deciduous teeth freely. In this there is less difficulty than with the permanent teeth.

I condemn the practice proposed by Dr. Arthur, in which he anticipates decay. A sound tooth should not be touched with the file. "The whole need not a physician." If the sound natural covering of healthy dentine is removed, decay will usually occur. The use of the file induces increased sensitiveness. This exalted sensitiveness is a pathological and not a physiological condition, and one in which active disease is very liable to set in.

Dr. Taylor: My attention has been much directed to this subject of exalted sensitiveness, and my opinion is that, though there be a high degree of exalted sensitiveness, when the decay with which it is connected is cut away by the file and the tooth smoothly polished, the decay is arrested and the surface solidified. I think the exalted sensitiveness indicates a condition that is favorable to this result.

Dr. Watt: Dr. McCullum, some years ago, settled the file question in a short paper. I think Dr. Arthur is certainly mistaken in his views upon the use of the file, especially as it regards the deciduous teeth, and more particularly sound teeth. The exalted sensitiveness referred to is a pathological condition, but that condition is many a time necessary in the process of reproduction of tissue, especially when there is great injury. In young patients the reproduction of tissue is much more under control and direction than in older persons. The file is a good instrument for trimming and dressing, but not for arresting decay. There is not always diminished action where there is disease, but many a time the action is above that of a healthy condition.

Dr. Morgan: Dr. Watt's theory is probably correct, still I do not understand how this pathological state in sensitive dentine can aid in the arrest of decay, or in the restoration of the diseased part to health.

Dr. Shadoan: Solidification of dentine does arrest decay; during this process doubtless there is an increase of vitality. All teeth are not alike susceptible to injury by the use of the file. This is a point we should study well. I do not approve of making large separations between the teeth with the file for filling.

Dr. Taylor: I do not file off ebernated surfaces, but regard that as an indication that the other teeth may be filed freely.

Second Subject—Treatment and preservation of the deciduous teeth.

Dr. Berry: I regard it as much the duty of the Dentist to instruct parents and those having the care of children, in reference to the proper means of taking care of their teeth, as to operate for them. Many are inclined to say just as little as possible upon this subject, lest they should be thought obtrusive.

Dr. H. A. Smith: The subject of the influence of the various kinds of food, for securing teeth of good structure, is an important one and should be well understood. How the file can be used to any extent upon the deciduous teeth I am at a loss to understand. It is contrary to my judgment and feelings. Dr. Morgan's experience is far more extensive than mine, and I must conclude is correct. Filling the teeth of children is a very important operation, and one that calls for much skill for its proper performance.

Dr. MORGAN: There is a very great want of appreciation and care of the temporary teeth by the Dental profession, nor have physicians given the subject the attention its importance demands. I have had an extended experience in the treatment of children's teeth, and I do not hesitate to operate freely upon them, both by filing and filling; am not so particular as to the material with which I fill as in the permanent teeth; fill with any thing that will last as long as the teeth are to remain. When an abscess occurs from a deciduous tooth, usually the latter should be removed; otherwise necrosis of the alveolus and destruction of the permanent tooth may be effected, and oftentimes is. Premature removal of the temporary teeth should be avoided, as it will render the proper eruption of the permanent teeth less certain and more difficult.

Dr. SHADOAN: Children often by accident have the temporary teeth loosened, yet have some attachment, but have inflammation and soreness about them and perhaps some discharge of pus.

Dr. MORGAN: It is very difficult to answer Dr. Shadoan's question. There are so many conditions, which he does not state, that must be taken into account. For instance, predisposition temperament and other conditions, that I can not now determine the best course of treatment. Could I see the case I would decide.

Dr. SHADOAN: I do not think the temporary teeth should be in all cases removed when they are luxated, though some Dentists invariably do it. When alveolar abscess occurs, I think the removal of the tooth is indicated.

Dr. MORGAN: A diseased deciduous tooth will often produce the same symptoms as difficult dentition. This will sometimes take place at two or three years of age.

Dr. J. H. PAINE: A child twenty two months of age, by a fall, had the two superior central incisors entirely displaced. After some forty minutes they were replaced and, being disposed to slip out of the sockets, they were ligated firmly in position, and in about six weeks were firmly fixed in the sockets and have continued to the present in a healthy condition.

Dr. McCULLUM: I regard light as a very important element in the development and preservation of all living tissues.

Dr. MORGAN: I do not regard the dark or greenish deposit so frequently found upon children's teeth like or analogous to salivary calculus, but it is a deposit of coloring matter upon a surface of decomposing enamel. In such cases I enjoin scrupulous cleanliness and frequent and thorough friction of the gums; have used the hypophosphates with marked advantage.

Dr. McCLELLAND: In such cases as Dr. Morgan has referred to I dress off and polish the rough surface very thoroughly, and then require strict cleanliness and the affection does not usually return.

Third Subject—Mechanical Dentistry.

[Introductory to the discussion of this subject, Prof. John Allen, of New York, read a paper upon "Continuous Gum Work," in the construction of artificial dentines for which see March number of the REGISTER. After the reading of the paper he gave a very full description and illustration of the present attainments in the construction of this work, by which it was clearly seen that progress is constantly being made.—REP.]

Dr. McCLELLAND gave a full description and demonstration, with implements and models, of the method of constructing artificial dentures upon Rose Pearl base, clearing up to the minds of the members many things that had been in doubt and uncertainty.

Dr. CAMERON: I have been using Rose Pearl base about four months; have made and put in the mouth about ten pieces, and in every case they are thus far successful and eminently satisfactory to both patient and myself. It is in every case much preferred to rubber. After understanding the method of working, it is about as easy to construct an artificial denture as by any other method, or with any other material.

Dr. H. A. SMITH: I am much pleased with Rose Pearl so far. I much prefer to work it than rubber. Should like to see it more extensively used by the profession.

Dr. McCLELLAND: A misfit is more easily corrected in the Rose Pearl than in any other kind of work. The surface of a plate may be softened and new material added to any required extent, and the attachment is most perfect.

[To be Continued.]

